# Retracted: Essential Oils Loaded in Nanosystems: A Developing Strategy for a Successful Therapeutic Approach

**DOI:** 10.1155/2021/7259208

**Published:** 2021-02-15

**Authors:** Evidence-Based Complementary and Alternative Medicine

*Evidence-Based Complementary and Alternative Medicine* has retracted the article titled “Essential Oils Loaded in Nanosystems: A Developing Strategy for a Successful Therapeutic Approach” [1] due to high level of text similarity with previously published articles [2–6], of which [2, 4] were not cited.

A reassessment of the article concluded that large areas of overlapping text were identified throughout the article, especially in Section 5, “EO-Loaded Nanodelivery Systems.” The article is therefore being retracted with the agreement of the editorial board and the authors.
